# Prognosefaktoren für das Gesamtüberleben bei Oropharynxkarzinomen in Abhängigkeit vom HPV-Status

**DOI:** 10.1007/s00106-021-01076-3

**Published:** 2021-06-25

**Authors:** A. Riders, M. Oberste, B. Abbaspour, A. Beule, C. Rudack

**Affiliations:** Klinik für Hals-Nasen-Ohrenheilkunde, Universitätsklinikum Münster, Kardinal-von-Galen-Ring 10, 48149 Münster, Deutschland

**Keywords:** Humanes Papillomavirus, Oropharynxkarzinom, Alkoholkonsum, Nikotinkonsum, Gesamtüberleben, Human papillomavirus, Oropharyngeal cancer, Nicotine consumption, Alcohol consumption, Overall survival

## Abstract

**Hintergrund:**

Aufgrund der unterschiedlichen Karzinogenese, Prognose und klinischen Manifestation werden seit der 8. Auflage des TNM-Klassifikationssystems der UICC/AJCC (UICC 8) humane Papillomavirus (HPV)-assoziierte und HPV-negative oropharyngeale Plattenepithelkarzinome (OSCC) als 2 Entitäten separat klassifiziert.

**Material und Methoden:**

Insgesamt 524 Patienten mit einem im Zeitraum von 2000–2016 in der HNO-Klinik des Universitätsklinikums Münster behandelten OSCC wurden hinsichtlich des Nachweises von HPV-Typ-16-spezifischer DNA (HPV16-DNA), des Nikotin- und Alkoholkonsums sowie des Therapieeinflusses auf das Gesamtüberleben (GÜ) untersucht.

**Ergebnisse:**

Ein signifikanter Anstieg der jährlichen Prävalenz der HPV16-DNA-positiven OSCC von 40 % (*n* = 12/30) im Jahr 2000 auf 46 % (*n* = 18/39) im Jahr 2016 wurde verzeichnet (*p* = 0,025, β = 0,539). 89 % (*n* = 212) der HPV16-DNA-positiven OSCC wurden anhand der UICC 8 gegenüber der UICC 7 herabgestuft. Im Gesamtkollektiv zeigten der häufige Alkohol- und Nikotinkonsum (≥ 10 Packungsjahre) einen statistisch relevanten negativen Einfluss auf das GÜ (*p* = 0,004 und *p* = 0,009). Auch häufiger Alkoholkonsum war in der HPV16-DNA-negativen Gruppe prognoserelevant (*p* = 0,049). In der HPV16-DNA-positiven Gruppe zeigte sich bezüglich des GÜ zwischen den UICC-Stadien I und II (*p* = 0,481) sowie zwischen III und IV (*p* = 0,439) gemäß UICC 8 kein statistischer Unterschied.

**Schlussfolgerungen:**

Die UICC 8 verbessert zwar die Prognosestratifikation der OSCC durch die Trennung von HPV-positiven und HPV-negativen Tumoren im Vergleich zu UICC 7, die prognostische Aussagekraft der UICC 8 für die HPV-assoziierten OSCC ist jedoch weiterhin unzureichend. Der Noxenkonsum könnte zukünftig Einfluss auf die UICC-Klassifikation nehmen, um die prognostische Aussagekraft weiter zu verbessern.

## Abstract (en)

**Background:**

Due to their differing carcinogenesis, prognosis and clinical manifestation, human papillomavirus (HPV)-associated and HPV-negative oropharyngeal squamous cell carcinoma (OSCC) have been classified separately as two entities since the 8th edition of the AJCC/UICC TNM staging system (UICC 8).

**Materials and methods:**

A total of 524 patients with OSCC treated between 2000 and 2016 at the Department of Otorhinolaryngology, Head and Neck Surgery of the University Hospital Muenster, Germany, were examined for the detection of HPV type 16-specific DNA (HPV16-DNA), nicotine and alcohol consumption and the influence of therapy on overall survival (OS).

**Results:**

There was a significant increase in the annual prevalence of HPV16-DNA-positive OSCC from 40% (*n* = 12/30) in 2000 to 46% (*n* = 18/39) in 2016 (*p* = 0.025, β = 0.539). Of the HPV16-DNA-positive OSCC, 89% (*n* = 212) were downgraded on the basis of UICC 8 compared to UICC 7. In the overall collective, frequent alcohol and nicotine (≥ 10 pack years) consumption showed a significant negative influence on OS (*p* = 0.004 and *p* = 0.009, respectively). Frequent alcohol consumption was also prognostically relevant in the HPV16-DNA-negative group (*p* = 0.049). In the HPV16-DNA-positive group, the prognosis for OS according to UICC 8 showed no statistically significant difference between stages I and II (*p* = 0,481), or between III and IV (*p* = 0.439).

**Conclusions:**

The current UICC 8 improves the prognostic stratification of OSCC due to the separation of HPV-positive and HPV-negative tumors in comparison to UICC 7. However, the prognostic significance of UICC 8 for HPV-associated OSCC is still insufficient. In the future, alcohol and nicotine consumption could influence the UICC classification in order to further improve prognostic significance.

Momentan steht die SARS-CoV-2(„severe acute respiratory syndrome coronavirus type 2“)-Pandemie weltweit im Mittelpunkt. Doch auch andere viral ausgelöste Erkrankungen verbreiten sich in unserer Bevölkerung mit schwerwiegenden Folgen. Dazu zählt die Infektion mit dem humanen Papillomavirus (HPV), die in den letzten Jahrzehnten immer mehr an Bedeutung gewonnen hat. Für die Entstehung der Plattenepithelkarzinome des Oropharynx (OSCC) spielen vor allem der High-risk-HPV-Typ 16 (HPV16) sowie Nikotin- und Alkoholabusus die maßgebliche Rolle.

Während die Inzidenzraten der Kopf-Hals-Karzinome parallel mit der Abnahme des Tabakkonsums insgesamt rückläufig sind, wird weltweit ein Anstieg der HPV-positiven OSCC mit deutlichen geographischen Unterschieden verzeichnet [12, 24]. Aufgrund der unterschiedlichen Karzinogenese und Prognose werden die HPV-positiven und -negativen OSCC seit 2017 in der UICC 8 (Union Internationale contre le Cancer) als separate Entitäten klassifiziert. Mehrere europäische Studien berichten jedoch über eine unzureichende Prognosestratifikation der HPV-positiven OSCC zwischen einzelnen Stadien der UICC 8 [6, 19, 27]. Der Einschluss weiterer Prognosefaktoren neben HPV und TNM könnte diese optimieren.

Aktuell werden in zahlreichen klinischen Studien Strategien von spezifischen, zielgerichteten sowie deintensivierten Therapien bei HPV-positiven OSCC untersucht, um eine verbesserte Lebensqualität ohne Verschlechterung der Prognose zu erreichen [15]. Zwei große multizentrischen Studien [9, 16], die den Ersatz einer platinbasierten Chemotherapie durch eine Antikörpertherapie mit Cetuximab bei simultaner Radiotherapie verglichen, konnten jedoch keine Vorteile im Therapiearm mit Cetuximab in der Behandlung HPV-induzierter OSCC herausstellen.

Das Ziel der vorliegenden Studie war die Überprüfung ausgewählter Prognosefaktoren, wie Nikotin, Alkohol und der Therapie in Abhängigkeit des Nachweises von HPV16-spezifischer DNA (HPV16-DNA) im Tumorgewebe, sowie die Analyse der Risikostratifizierung der UICC 8 bezogen auf das Gesamtüberleben (GÜ) in dem vorliegendem primär chirurgisch behandelten Patientenkollektiv.

## Material und Methoden

Insgesamt wurden 524 Patienten mit der Erstdiagnose eines OSCC berücksichtigt, die in der HNO-Klinik des Universitätsklinikums Münster im Zeitraum von 2000–2016 behandelt wurden. Es lagen Genehmigungen der Ethikkommissionen der Ärztekammer Westfalen-Lippe und der Westfälischen Wilhelms-Universität (2013-511-f-S) vor. Die Daten wurden anhand der digitalen Krankenakten akquiriert und pseudonymisiert. Der Beobachtungszeitraum erstreckte sich vom Zeitpunkt der Erstdiagnose bis zum letzten Tumornachsorgetermin bzw. bis zum Versterben des Patienten.

Alkoholkonsum an mindestens 4 Tagen in der Woche wurde als häufiger Alkoholkonsum klassifiziert. Patienten mit einem Nikotinkonsum ≥ 10 Packungsjahre (PY) wurden als intensive Raucher charakterisiert. Die Therapie wurde in folgenden Kategorien klassifiziert:

- alleinige Tumoroperation (OP; *n* = 42),
- Operation und adjuvante Radiatio (OP + RT; *n* = 139),
- Operation und adjuvante Radiochemotherapie (OP + RCT; *n* = 146) und
- primäre Radiochemotherapie (RCT; *n* = 92).

Für die Überlebensanalyse hinsichtlich der Therapie wurden die Patienten mit vollendeter primärer kurativer Therapie und von den operierten Patienten (*n* = 327) nur diejenigen mit sicheren R0-Resektionen (*n* = 251) eingeschlossen, um mögliche Verzerrungen wegen adjuvanter Therapieeskalation bei einem positiven R‑Status zu vermeiden. Bei vorhandenen Risikofaktoren, wie nodaler Metastasierung, kapselüberschreitendem Wachstum, knappen tumorfreien oder positiven Resektionsrändern, wurde durch die postoperative Tumorkonferenz die Indikation für eine adjuvante Therapie gestellt. Standardmäßig erfolgte eine Strahlentherapie bei primärer Therapie mit einer Strahlendosis bis 72 Gy und im adjuvanten Setting bis 66 Gy über 6 Wochen. Die Chemotherapie erfolgte in erster Linie mit Cisplatin (bis 2011: 2–3 × 100 mg/m^2^, ab 2012: 5–6 × 40 mg/m^2^).

### Nachweis von HPV16-DNA

Das im Rahmen der vorliegenden Studie verwendete Gewebe entstammte einem diagnostischen Routineeingriff und wurde neben der standardisierten Histopathologie des Gerhard-Domagk-Instituts für Pathologie Münster auf HPV16-DNA untersucht. Die formalinfixierten, paraffineingebetteten Gewebestücke wurden zunächst in 20 µm dünne Schichten geschnitten und entparaffiniert. Im Anschluss wurde die DNA mittels eines kommerziellen Kits (QIAamp DNA Mini Kit, QIAGEN, Hilden) gemäß dem Protokoll des Herstellers isoliert. Humanes β‑Globin-Gen wurde als Indikator für eine erfolgreiche Extraktion einer ausreichend großen Menge DNA angewendet. Anschließend wurden HPV16-DNA der Onkogene E6 und E7 mittels Echtzeit-Polymerasekettenreaktion (ABI Prism 7900HT Sequenzerkennungssystem, TaqMan Genotyping PCR Master Mix, Applied Biosystems, Darmstadt, Deutschland) amplifiziert. Aus der ca. 600 Kopien im Genom integrierte HPV16-DNA enthaltenden humanen Zervixkarzinomzelllinie CaSki (ATCC® CRL-1550™, Mananssas, VA, USA) isolierte DNA wurde als positive Kontrolle genutzt. Von Tonsillengewebe eines 4‑jährigen Kindes nach einer Tonsillotomie isolierte DNA wurde als negative Kontrolle angewendet.

### Statistische Analyse

Die gesammelten Daten wurden mithilfe des Programms SPSS (IBM SPSS 26.0) analysiert. Zur Überprüfung der statistischen Unterschiede bezüglich der Häufigkeitsverteilung der HPV16-DNA-positiven und -negativen Gruppen wurde der Pearson χ^2^-Test angewandt, zum Vergleich des medianen Lebensalters der Mann-Whitney-U-Test. Um die Prävalenzveränderungen im beobachteten Zeitraum zu überprüfen, erfolgte eine lineare Regressionsanalyse. Für die Überlebensanalyse wurden die Kaplan-Meier-Methode und der Log-Rank-Test angewendet. Als Signifikanzniveau wurde *p* ≤ 0,05 festgelegt.

## Ergebnisse

### Patienten- und Tumorcharakteristika

**Figure 1:**
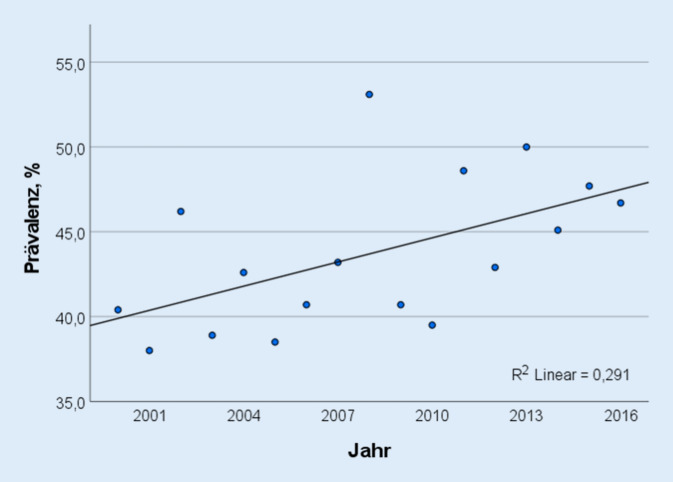

Im vorliegenden Gesamtkollektiv betrug der Anteil der HPV16-DNA-positiven OSCC 45,4 % (*n* = 238/524). Außerdem wurde ein signifikanter Anstieg der jährlichen Prävalenz der HPV16-DNA-positiven OSCC von 40 % (*n* = 12/30) im Jahr 2000 auf 46 % (*n* = 18/39) im Jahr 2016 verzeichnet (*p* = 0,025; β = 0,539; Abb. 1). Hinsichtlich des medianen Lebensalters der Patienten zum Zeitpunkt der Diagnosestellung zeigte sich kein statistischer Unterschied zwischen beiden Gruppen (60,7 ± 9,8 vs. 60,3 ± 10,2 Jahre, *p* = 0,161; Tab. 1). Die HPV16-DNA-positiven OSCC wiesen bei Erstdiagnose häufiger einen kleineren Primärtumor (T1–T2: 68,9 % [*n* = 164] vs. 54,1 % [*n* = 155], *p* = 0,001) und eine lokale Metastasierung (N1–N3: 81,2 % [*n* = 195] vs. 68,5 % [*n* = 196], *p* = 0,001; Tab. 1) im Vergleich zu HPV16-DNA-negativen OSCC auf. Das Auftreten von Fernmetastasen bei Erstdiagnose war nicht vom HPV16-DNA-Status abhängig (M1: 4,6 % (*n* = 11) vs. 7,0 % (*n* = 20), *p* = 0,337; Tab. 1). Die Patienten mit einem HPV16-DNA-positiven OSCC wiesen signifikant seltener einen kumulativen Nikotinkonsum ≥ 10PY (63,0 % [*n* = 150] vs. 84,6 % [*n* = 242], *p* < 0,001) und häufigen Alkoholkonsum (31,1 % [*n* = 74] vs. 56,6 % [*n* = 162], *p* < 0,001) auf. In der HPV16-DNA-positiven Gruppe wurde signifikant häufiger eine adjuvante RCT verabreicht (41,2 % [*n* = 82] vs. 29,1 % [*n* = 64], *p* = 0,006; Tab. 1). Die Patienten, die einer primären RCT zugeführt wurden, zeigten häufiger ein höheres UICC-Stadium im Vergleich zu operierten Patienten sowohl in der HPV16-DNA-negativen (UICC-8-Stadien III–IV: 93,2 % [*n* = 55] vs. 67,1 % [*n* = 108], *p* = 0,001) als auch in der HPV16-DNA-positiven Gruppe (UICC-8-Stadien III–IV: 45,5 % [*n* = 15] vs. 7,2 % [*n* = 12], *p* = 0,001; Tab. 1).

**Table 1:** 

*Parameter*		*HPV16-DNA-negativ* *n* *=* *286 (54,6* *%)*			*HPV16-DNA-positiv* *n* *=* *238 (45,4* *%)*		*p‑Wert*
*Alter (Jahre)*		60,7 ± 9,8			60,3 ± 10,2		*p* = 0,161
–		*n (%)*			*n (%)*		–
*Geschlecht*	*Weiblich*	64 (22,5)			66 (27,6)		*p* = 0,187
	*Männlich*	222 (77,6)			172 (72,2)		
*T‑Status*	*T1–T2*	155 (54,1)			164 (68,9)		***p*** **=** **0,001**
	*T3–T4*	131 (45,8)			74 (31,0)		
*N‑Status*	*N0*	90 (31,5)			43 (18,0)		***p*** **=** **0,001**
	*N1–N3*	196 (68,5)			195 (81,2)		
*M‑Status*	*M0*	266 (93,0)			227 (95,4)		*p* = 0,337
	*M1*	20 (7,0)			11 (4,6)		
*Nikotinkonsum*	*<* *10PY*	44 (15,3)			88 (36,9)		***p*** **<** **0,001**
	*≥* *10PY*	242 (84,6)			150 (63,0)		
*Alkoholkonsum*	*Kein/gelegentlich*	124 (43,4)			164 (68,9)		***p*** **<** **0,001**
	*Häufig*	162 (56,6)			74 (31,1)		
*Therapie*	*Alle*	*OP*	25 (11,4)		17 (8,5)		***p*** **=** **0,017**
		*OP* *+* *RT*	72 (32,7)		67 (33,7)		
		*OP* *+* *RCT*	64 (29,1)		82 (41,2)		
		*Primäre RCT*	59 (26,8)		33 (16,6)		
	*OP* *+* *RCT vs. andere Therapie*	*OP* *+* *RCT*	64 (29,1)		82 (41,2)		***p*** **=** **0,006**
		*Andere Therapie*	156 (70,1)		117 (58,8)		
–		*HPV16-DNA-negativ*			*HPV16-DNA-positiv*		
		*OP* *±* *R(C)T* *n (%)*	*Primäre RCT* *n (%)*	*p‑Wert*	*OP* *±* *R(C)T* *n (%)*	*Primäre RCT* *n (%)*	*p‑Wert*
*UICC 8*	*I–II*	53 (32,9)	4 (6,6)	***p*** **<** **0,001**	154 (92,8)	18 (54,5)	***p*** **<** **0,001**
	*III–IV*	108 (67,1)	55 (93,2)		12 (7,2)	15 (45,5)	

*OP* Operation, *RT* Radiotherapie, *RCT* Radiochemotherapie, *R(C)T* Radiatio ± Chemotherapie, *UICC* Union Internationale contre le Cancer

### Univariate Gesamtüberlebensanalyse

**Figure 2:**
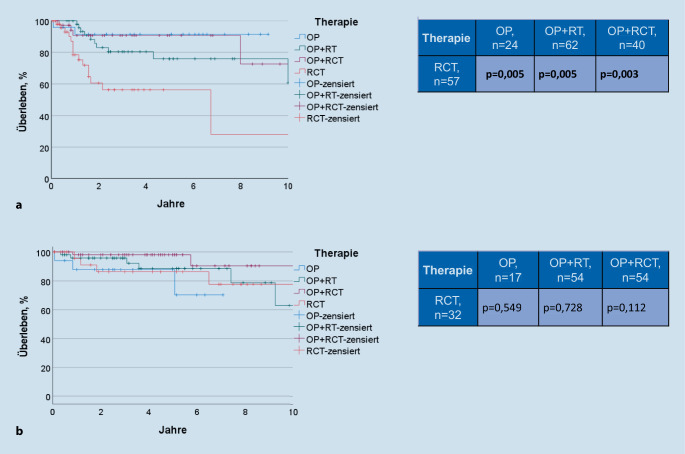

Patienten mit einem HPV16-DNA-positiven OSCC zeigten eine um 18,5 % bessere 5‑Jahres-Gesamtüberlebensrate (5-JGÜ) als die Patienten mit einem HPV16-DNA-negativen OSCC (88,3 vs. 69,8 %, *p* < 0,001; Tab. 2). Im Gesamtkollektiv zeigten häufiger Alkoholkonsum (*p* = 0,004) und Nikotinkonsum ≥ 10PY (*p* = 0,009; Tab. 2) einen signifikanten negativen Einfluss auf das GÜ. Wurde der Einfluss auf das GÜ in Abhängigkeit des HPV16-DNA-Status vergleichend betrachtet, ergab sich jedoch ein statistisch negativer Einfluss von häufigem Alkoholkonsum auf das GÜ nur bei Patienten mit HPV16-DNA-negativem OSCC (*p* = 0,049). Die Patienten mit Nikotinkonsum ≥ 10PY wiesen ein schlechteres GÜ sowohl in der HPV16-DNA-negativen als auch in der HPV16-DNA-positiven Gruppe auf, aber es ergab sich keine statistische Signifikanz (*p* = 0,322 und *p* = 0,095; Tab. 2). Innerhalb der HPV16-DNA-negativen Gruppe wiesen die Patienten, die eine primäre RCT (5-JGÜ 56,3 %) erhalten hatten, ein signifikant schlechteres GÜ im Vergleich zu Patienten nach OP, OP + RT und OP + RCT auf (5-JGÜ 91,5 %, *p* = 0,005; 75,9 %, *p* = 0,005 und 90,7 %, *p* = 0,003; Tab. 2; Abb. 2). Innerhalb der HPV16-DNA-positiven Gruppe zeigten die Patienten nach primärer RCT (5-JGÜ 86,3 %) wiederum keinen signifikanten Unterschied des GÜ im Vergleich zu Patienten nach OP, OP + RT und OP + RCT (5-JGÜ 87,8 %, *p* = 0,549; 88,6 %, *p* = 0,728 und 98,0 %, *p* = 0,112; Tab. 2; Abb. 2).

**Table 2:** 

Parameter		Gesamtkollektiv			HPV16-DNA-negativ			HPV16-DNA-positiv		
		*n* (%)	5‑JGÜ (%)	*p*-Wert	*n* (%)	5‑JGÜ, (%)	*p*-Wert	*n* (%)	5‑JGÜ (%)	*p*-Wert
HPV16-DNA-Status	Negativ	286 (54,6)	69,8	*p* < 0,001	–					
	Positiv	238 (45,4)	88,3							
Geschlecht	Weiblich	130 (24,8)	87,1	*p* = 0,073	64 (22,5)	80,4	*p* = 0,087	66 (27,6)	90,9	*p* = 0,775
	Männlich	394 (75,2)	76,4		222 (77,6)	66,9		172 (72,2)	86,9	
Nikotinkonsum (PY)	< 10	132 (25,2)	87,2	*p* = 0,009	44 (15,3)	78,9	*p* = 0,322	88 (36,9)	90,7	*p* = 0,095
	≥ 10	392 (74,8)	75,7		242 (84,6)	68,3		150 (63,0)	85,9	
Alkoholkonsum	Kein/gelegentlich	288 (55,0)	84,2	*p* = 0,004	124 (43,4)	79,1	*p* = 0,049	164 (68,9)	87,7	*p* = 0,539
	Häufig	236 (45,0)	70,6		162 (56,6)	63,0		74 (31,1)	86,2	
Therapie	OP	41 (12,1)	90,0	*p* = 0,001	24 (13,2)	91,5	*p* = 0,001	17 (10,8)	87,8	*p* = 0,160
	OP + RT	116 (34,2)	82,3		62 (34,1)	75,9		54 (34,4)	88,6	
	OP + RCT	94 (27,7)	95,2		40 (22,0)	90,7		54 (34,4)	98,0	
	Primäre RCT	89 (26,1)	68,9		57 (31,1)	56,3		32 (20,4)	86,3	

*5‑JGÜ* 5-Jahres-Gesamtüberlebensrate, *OP* Operation, *PY* „pack years“, *RT* Radiotherapie, *RCT* Radiochemotherapie

### Stratifikation gemäß UICC 7 und UICC 8

**Figure 3:**
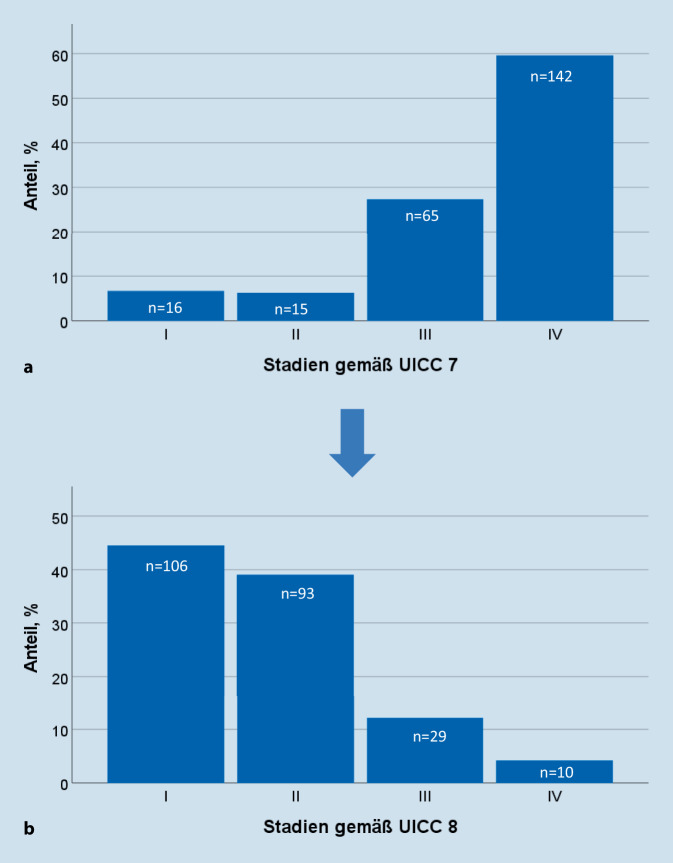

**Figure 4:**
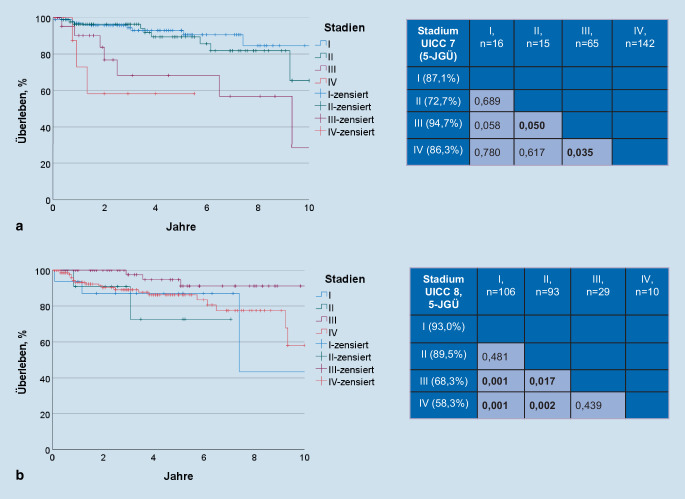

Neunundachtzig Prozent (*n* = 212) der HPV16-DNA-positiven OSCC wurden anhand der UICC 8 gegenüber der UICC 7 einem niedrigeren Stadium zugeordnet. Gemäß UICC 7 befand sich der Großteil der HPV16-DNA-positiven OSCC in Stadien IV (59,7 %, *n* = 142) und III (27,3 %, *n* = 65; Abb. 3). Gemäß UICC 8 befanden sich demgegenüber wiederum mehr HPV16-DNA-positive OSCC in den Stadien I (44,5 %, *n* = 106) und II (39,1 %, *n* = 93). In der HPV16-DNA-positiven Gruppe zeigte sich gemäß UICC 7 ein signifikant unterschiedliches GÜ lediglich zwischen den Stadien II und III (*p* = 0,050) sowie III und IV (*p* = 0,035; Abb. 4). Außerdem wiesen die Patienten mit dem UICC-7-Stadium III paradoxerweise ein signifikant günstigeres GÜ als die Patienten mit Stadium II (5-JGÜ 94,7 vs. 72,7 %, *p* = 0,050; Abb. 4) auf. Nach der Re-Klassifizierung gemäß UICC 8 ergab sich eine signifikante Prognosestratifikation zwischen den Stadienpaaren I und III, I und IV, II und III, sowie II und IV (*p* = 0,001; *p* = 0,001; *p* = 0,017; *p* = 0,002; Abb. 4). Es bestand jedoch weiterhin kein signifikanter Unterschied des GÜ zwischen den UICC-8-Stadien I und II (*p* = 0,481) sowie zwischen III und IV (*p* = 0,439).

## Diskussion

### Besseres Gesamtüberleben und steigende Prävalenz

Eine günstigere Prognose der Patienten mit einem HPV16-DNA-positiven OSCC trotz der erhöhten lokalen Metastasierungsrate steht im Einklang mit der Literatur [1, 6, 25]. Der Anstieg der jährlichen Prävalenz der HPV16-DNA-positiven OSCC zwischen 2000–2016 bestätigt ebenfalls den allgemeinen Konsens über die zunehmende Verbreitung der HPV-positiven OSCC in den Industrieländern [3, 14, 24]. Die Gründe dafür sind jedoch noch nicht vollständig geklärt [25]. Obwohl in mehreren Studien ein verändertes Sexualverhalten als mögliche Ursache hierfür diskutiert wird [3–5], konnte eine aktuelle Studie aus Deutschland keine Korrelation zwischen sexuellen Präferenzen und der HPV-Infektion zeigen [20].

### Regionale Altersunterschiede

Die vorliegenden Daten zeigten keinen statistisch signifikanten Altersunterschied zwischen Patienten mit einem HPV16-DNA-positiven und -negativen OSCC. Mehrere Publikationen aus Deutschland beschreiben die Patienten mit einem HPV-assoziierten OSCC ebenfalls als gleichaltrig mit [1, 27] oder sogar älter als [23] Patienten mit einem HPV-negativen OSCC. Zahlreiche US-amerikanischen Studien weisen wiederum auf ein jüngeres Alter bei Patienten mit einem HPV-positiven OSCC hin [2, 22].

### Nikotin- und Alkoholabusus

Analog zu einer vergleichbaren Studie aus Deutschland [27] ergab sich der Alkoholkonsum als ein signifikanter Prognosefaktor innerhalb des Gesamtkollektivs und in der HPV-negativen Gruppe. In einer anderen Publikation zeigten Würdemann et al. einen statistisch negativen Einfluss von häufigem Alkoholkonsum auf das GÜ bei Patienten mit einem HPV-positiven wie mit einem HPV-negativen OSCC [26]. Sowohl in der vorliegenden Untersuchung als auch in einer Publikation aus Deutschland [27] zeigte ein Tabakkonsum ≥ 10PY eine statistisch signifikante Assoziation zu schlechterem GÜ hinsichtlich des gesamten Patientenkollektivs mit OSCC und keinen statistisch signifikanten Einfluss auf das GÜ in der HPV-positiven und -negativen Gruppe. Die vorliegenden Ergebnisse bestätigen die Literaturdaten, dass die Patienten mit einem HPV-positiven OSCC eine niedrigere Exposition gegenüber Nikotin und Alkohol aufweisen als Patienten mit einem HPV-negativen OSCC [1, 6, 10, 11, 18, 27]. Niedrigerer Noxenkonsum ist mit einem besseren Gesundheitszustand und mit weniger Komorbiditäten vergesellschaftet [1, 7] und erklärt zum Teil den Überlebensvorteil der Patienten mit einem HPV-positiven OSCC [11]. Die Patienten sollten jedoch ermutigt werden, auch nach der Erstdiagnose den Nikotinkonsum zumindest zu reduzieren, da eine aktuelle Studie aus Deutschland zeigte, dass die Reduktion oder die komplette Einstellung des Tabakkonsums die Prognose verbessert [8].

### Prognosestratifikation gemäß UICC 8 noch nicht optimal

In dem hier vorgestellten Patientenkollektiv spiegelt die UICC 8 die Prognose der Patienten mit HPV-assoziiertem OSCC im Vergleich zu UICC 7 besser wider. Es besteht jedoch weiterhin kein statistischer Unterschied der Prognose zwischen den UICC-8-Stadien I und II sowie III und IV. Da die absolute Mehrheit der Patienten mit HPV-assoziiertem OSCC zu den UICC-8-Stadien I und II gehören, erscheint die prognostische Aussagekraft der aktualisierten TNM-Klassifikation weiterhin unzureichend. Vergleichbare europäische Studien zeigten hinsichtlich der HPV-positiven OSCC keinen statistischen Unterschied der Prognosestratifikation zwischen den UICC-8-Stadien II und III [6, 27] sowie I und II [19]. Daher scheinen weitere Anpassungen erforderlich, um die Vorhersage der Überlebensprognose zu verbessern. Die Hinzunahme weiterer Prognosefaktoren in die TNM-Klassifikation könnte möglicherweise die Risikostratifizierung optimieren.

### Therapiebezogene Unterschiede in Abhängigkeit vom HPV-Status

Konkordant zur Literatur [1] ergab auch die vorliegende Untersuchung, dass die Patienten mit einem HPV16-DNA-positiven OSCC nach Operation statistisch häufiger eine adjuvante RCT erhielten. Dieses Phänomen ist einerseits erklärbar durch die erhöhte Neigung zu einer lokalen Metastasierung der HPV-positiven OSCC, weshalb die HPV-positiven OSCC anhand der TNM 7 einem höheren N‑Status zugeordnet wurden. Andererseits gibt es Hinweise, dass der oft durch den reduzierten Noxenkonsum bedingte bessere Allgemeinzustand mit weniger Begleiterkrankungen der Patienten mit einem HPV-positiven OSCC eine adjuvante RCT häufiger ermöglicht bei sonst analoger Risikokonstellation im Vergleich zu Patienten mit einem HPV-negativen OSCC [1]. In einer anderen Studie aus Deutschland zeigten Nikotinabusus und eine höhere Anzahl an Komorbiditäten wiederum keinen negativen Einfluss auf die Dosiserreichung von R(C)T (Radiatio ± Chemotherapie) bei Patienten mit Kopf-Hals-Karzinomen [7].

Passend zur klinischen Erfahrung, dass in Deutschland typischerweise bei operablem OSCC die chirurgische Therapie bevorzugt wird, zeigten sowohl Patienten mit einem HPV16-DNA-negativen als auch Patienten mit HPV16DNA-positiven OSCC, die einer primären RCT zugeführt wurden, einen größeren Anteil an höheren UICC-Stadien. Während innerhalb der HPV16-DNA-negativen Gruppe die Patienten nach primärer RCT ein schlechteres GÜ im Vergleich zu operierten Patienten – OP ± R(C)T – zeigten, wiesen die Patienten mit einem HPV16-DNA-positiven OSCC nach primärer RCT wiederum eine mit der von operierten Patienten vergleichbare Prognose auf. Auch eine ähnliche retrospektive Studie aus Frankreich liefert Hinweise, dass die HPV-assoziierten OSCC besser auf eine RCT ansprechen als HPV-negative OSCC. Während im Gesamtkollektiv die Patienten mit OSCC nach primärer RCT ein schlechteres krankheitsfreies Überleben im Vergleich zu Patienten nach OP ± R(C)T zeigten, wiesen die Patienten mit einem p16-positiven OSCC eine vergleichbare Prognose nach OP ± R(C)T und primärer RCT auf [17]. Obwohl man in der Literatur widersprüchliche Ergebnisse findet, zeigt die Mehrheit der Studien eine höhere Sensitivität der HPV-positiven Tumorzellen auf eine Radiatio sowohl in vivo [13] als auch unter In-vitro-Bedingungen [21].

## Fazit für die Praxis

- In Deutschland und anderen Industrieländern wird ein Prävalenzanstieg der HPV-positiven OSCC verzeichnet.
- Die aktualisierte UICC 8 verbessert zwar die Prognosestratifikation der Patienten mit einem HPV-positiven OSCC im Vergleich zu UICC 7, ist jedoch noch nicht optimal.
- Erhöhter Noxenkonsum und assoziierte Komorbiditäten sind prognoserelevant für Patienten mit OSCC, daher könnten diese Faktoren zukünftig Einfluss auf die UICC-Klassifikation nehmen.
- Obwohl es Hinweise auf eine erhöhte Strahlenempfindlichkeit der HPV-positiven OSCC gibt, bestehen derzeit in der Praxis noch keine Unterschiede im Behandlungskonzept der OSCC in Abhängigkeit vom HPV-Status. Es ist jedoch davon auszugehen, dass in Zukunft für bestimmte OSCC-Patientengruppen mit niedrigem Risiko eine Therapiedeeskalation ermöglicht wird.
